# Evidence of effective cardiovascular countermeasures during spaceflights: insights from wearable monitoring

**DOI:** 10.1038/s41526-025-00522-8

**Published:** 2025-09-30

**Authors:** Paniz Balali, Elena Luchitskaya, Amin Hossein, Elza Abdessater, Vitalie Faoro, Olivier Debeir, Jens Tank, Enrico Gianluca Caiani, Philippe van de Borne, Pierre-François Migeotte, Jeremy Rabineau

**Affiliations:** 1 Laboratory of Physics and Physiology (LPHYS), Département de Cardiologie, Hôpital universitaire d’Erasme, Université libre de Bruxelles, Brussels, Belgium; 2 Laboratory of Image Synthesis and Analysis (LISA), Université Libre de Bruxelles, Brussels, Belgium; 3 Institute of Biomedical Problems (IBMP), Russian Academy of Sciences, Moscow, Russian Federation; 4 Research Unit in Cardio-respiratory Physiology, Exercise & Nutrition, Faculty of Human Movement Sciences, Université libre de Bruxelles, Brussels, Belgium; 5 Institute of Aerospace Medicine, German Aerospace Center (DLR), Cologne, Germany; 6 Electronic, Information and Biomedical Engineering Department, Politecnico di Milano, Milan, Italy; 7 IRCCS Istituto Auxologico Italiano, Milan, Italy; 8 Département de Cardiologie, Université de Mons, Mons, Belgium; 9 Department of Kinesiology and Health Sciences, University of Waterloo, Waterloo, Ontario Canada; 10 Brussels Laboratory of the Universe - BLU-ULB, Université libre de Bruxelles, Brussels, Belgium

**Keywords:** Biomedical engineering, Physiology

## Abstract

Microgravity induces profound cardiovascular changes, prompting space agencies to develop countermeasures to preserve their crewmembers’ health. This study aimed to use a portable device based on electro-, impedance- and seismo-cardiography, to monitor a series of cardiovascular features in 17 cosmonauts. Our results showed that the evolution of cardiac time intervals, blood pressure, stroke volume, and cardiac systolic kinetic energy depended on the chosen baseline position. After five months in space, heart rate increased compared to the supine baseline on Earth (p = 0.013, d = 0.86) but not to the sitting position. Similarly, a marker of cardiac contractility (PEP/LVET ratio) decreased relative to the sitting baseline (p = 0.004, d = 1.09) but not the supine reference. All measured features, except heart rate, returned to baseline within three days post-landing. These findings support the efficacy of current countermeasures in facilitating rapid cardiovascular re-adaptation to terrestrial gravity.

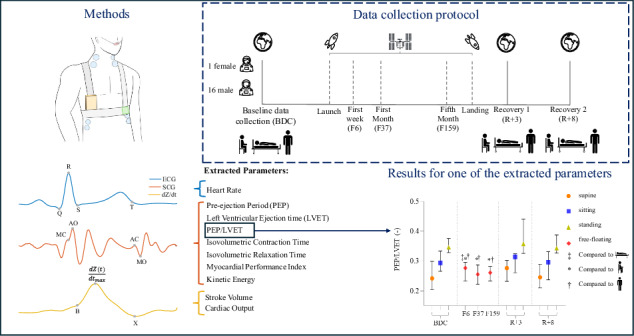

## Introduction

Exposure to microgravity induces physiological changes, notably in blood circulation. Upon entering microgravity, weightlessness triggers a redistribution of blood, causing an increased flow to the head while reducing circulation to the lower body^1^. In response to this fluid shift, the body initiates an adaptation process, reducing blood volume^2,3^. Typically, this adaptive process unfolds over several days, potentially leading to alterations in the cardiovascular system, particularly in the absence of countermeasures^4^.

In recent years, significant progress has been made in understanding cardiovascular adaptations during spaceflight. However, controversies persist regarding the cardiovascular changes during extended space missions, including for some basic features such as stroke volume (SV)^5–8^ and heart rate (HR)^7,9–11^, as highlighted in recent studies with inconsistent findings. These differing findings indicate the complexity and inter-individual differences of cardiovascular responses during space missions, emphasizing the need for further research to reconcile these differences.

Most of the observed hemodynamic changes do not represent a deconditioning to a pathological state, but a physiological adaptation to the microgravity environment that is not problematic as long as the subjects remain in microgravity^12^. However, the long-term effects of these adaptations remain largely unknown and understudied, as the cumulative impact of extended exposure to microgravity on cardiovascular health is poorly understood. This knowledge gap is particularly concerning given the potential implications for crew health and mission performance. Frequent and accurate cardiovascular monitoring is essential to address these uncertainties, enabling detailed and personalized follow-up.

However, certain limitations arise when considering the applicability of traditional diagnostic devices in space. While cardiac MRI and echocardiography stand as gold standard modalities for assessing cardiovascular function on Earth, their use in space is constrained by practical issues. The substantial size, weight, and magnetic field of MRI equipment prevent its use in space, and the need for specialized expertise in echocardiographic measurements poses challenges. While guided echocardiography from Earth may be feasible in low Earth orbit^13^, deep-space missions to the Moon and Mars require autonomous, lightweight solutions. Using wearable devices can be an alternative approach. Techniques like seismocardiography (SCG) and impedance cardiography (ICG) offer portable alternatives, with a potential to be used in space. SCG measures the mechanical vibrations on the surface of the chest that result from the heart’s contraction, providing insight into cardiac function^14^. ICG, on the other hand, measures the electrical conductivity of the thorax, which detects thoracic fluid content changes (basal impedance) together with blood flow during the cardiac cycle^15–17^. These signals are directly related to the mechanical and volumetric changes in the heart and central blood vessels, making them useful for cardiovascular monitoring.

Further highlighting the use of these wearable techniques, SCG has been employed to gather information on the contractility state^18^, arterial stiffness^19^, cardiac time intervals^20–24^, and cardiorespiratory fitness^25^. One of the key features derived from SCG is the kinetic energy transmitted to the chest by the cardiac contractions. This kinetic energy provides insights into the mechanical efficiency of the heart and can be used to monitor changes in cardiac function over time^26^. Additionally, SCG can be used to determine the timing of aortic and mitral valve events, thus enabling the calculation of parameters such as pre-ejection period (PEP), left ventricular ejection time (LVET), isovolumetric relaxation time (IVRT), and isovolumetric contraction time (IVCT)^23^. However, despite its characteristics making SCG well-suited for space measurements, its utilization in space has been limited, with only a few crew members involved, primarily as a proof of concept^27^. ICG, on the other hand, offers insights into SV and cardiac output (CO)^15,28^. Its ability to non-invasively monitor SV variations under different physiological conditions makes it an interesting tool for assessing cardiac efficiency^29^. Although ICG is not as accurate as invasive techniques, it can effectively track individual relative changes of thoracic fluid content and blood flow over time^30^.

As gravity fundamentally shapes hemodynamic responses on Earth, understanding the influence of body position on cardiovascular function is crucial in spaceflight studies. The position of the body on Earth (supine, sitting, and standing) has a significant impact on the different cardiovascular features^31^. The comparison of cardiovascular data measured in space with the same data measured on Earth is thus highly complicated because there is no terrestrial equivalent to the state of weightlessness. While the supine position is often used as a reference, it may be less characteristic of a cardiovascular status meant to represent daily life. Indeed, healthy individuals usually spend about one-third of their day in a supine position, while most spend more time in a sitting position^32^. Neglecting these factors complicates the interpretation and comparison of findings, underscoring the need for more detailed and controlled studies to better understand cardiovascular function in microgravity environments.

The aim of the present study is to test the hypothesis that, when appropriate countermeasures are properly applied during space flight, the hemodynamic status of crew members in space is comparable to the one observed on Earth. To investigate this, we compare hemodynamic changes across the supine, sitting, and standing positions on Earth with those occurring in space. These comparisons are conducted in a cohort of cosmonauts undergoing modern countermeasures while on the International Space Station (ISS), using advanced non-intrusive hemodynamic wearable monitoring technology including electrocardiogram (ECG), SCG, and ICG. Specifically, this article examines changes in cardiovascular function, including cardiac time intervals, SCG-derived kinetic energy, blood pressure, and ICG-derived SV on cosmonauts assigned to long-duration spaceflights.

## Results

### Cardiac time intervals

Cardiac time intervals were first compared to the sitting baseline position. Table 1 provides a detailed account of the main cardiac time intervals measured throughout the study period. Compared to the baseline sitting position, HR was reduced both during the first week (p = 0.003, d = 0.67) and the first month in space (p = 0.028, d = 0.60), but not after five months in space. During the initial week of exposure to microgravity, PEP, PEP normalized for HR (PEPi), and PEP/ LVET showed a decrease from baseline (p = 0.036, d = 0.59; p = 0.020, d = 0.75; and p = 0.016, d = 0.87, respectively), with these lower levels persisting up to the fifth month in space (p = 0.017, d = 0.68; p = 0.020, d = 0.73; and p = 0.004, d = 1.09, respectively). In contrast, LVET and LVET normalized for sex and HR (LVETi) did not exhibit significant changes compared to baseline during this period. Meanwhile, as shown in Table 1, the myocardial performance index (MPI) was decreased at all the three timepoints in microgravity (p = 0.013, d = 0.88; p = 0.008, d = 0.93; and p = 0.001, d = 0.93 after one week, one month, and five months, respectively).

**Table 1:** Evolution of the features based on cardiac time intervals throughout the study

	BDC sitting n = 17	F6 n = 17	F37 n = 17	F159 n = 17	R + 3 sitting n = 15	R + 8 sitting n = 17
HR (bpm)	66 [61,75]	61 [56,67]*	62 [54,68]*	61 [60,69]	79 [70,81]*	71 [67,76]*
PEP (ms)	95 [83,99]	87 [81,95]*	88 [75,97]*	85 [79,95]*	87 [80,100]	87 [77, 105]
PEPi (-)	117 [113, 125]	111 [106, 120]*	112 [101, 121]*	114 [120, 106]*	118 [110, 130]	111 [107, 134]
LVET (ms)	304 [286, 330]	324 [312, 348]	327 [318, 357]	334 [318, 359]	334 [318, 359]	300 [271, 316]
LVETi (-)	421 [409, 438]	427 [416, 448]	436 [418, 476]	438 [424, 467]	429 [402, 438]	429 [404, 446]
PEP/LVET (-)	0.29 [0.26, 0.33]	0.27 [0.23, 0.29]*	0.25 [0.22, 0.28]*	0.26 [0.23, 0.28]*	0.31 [0.26, 0.32]	0.29 [0.23, 0.33]
IVCT (ms)	59 [55,71]	60 [50,65]	52 [49,68]	53 [51,66]	55 [53,63]	52 [48,61]
IVRT (ms)	26 [24,30]	22 [20,26]	23 [20,25]	24 [20,28]	26 [18,31]	29 [22,32]
MPI (-)	0.29 [0.24, 0.35]	0.25 [0.21, 0.28]*	0.23 [0.20, 0.27]*	0.24 [0.21, 0.28]*	0.28 [0.23, 0.33]	0.27 [0.23, 0.32]

Measurements are taken at baseline (BDC), during the flight at day 6 ± 2, 37 ± 4, and 159 ± 4 (F6, F37, and F159, respectively), as well as during recovery at 3 and 8 days post-flight (R + 3 and R + 8, respectively). Comparisons are made relative to baseline values in the sitting position using a linear mixed-effects model with Holm-Sidak correction for multiple comparisons. Results are presented as median [first quartile; third quartile].

*HR* heart rate, *PEP* pre-ejection period, *PEPi* pre-ejection period index (-), *LVET* left ventricular ejection time, *LVETi* left ventricular ejection time index, *IVCT* isovolumetric contraction time, *IVRT* isovolumetric relaxation time, *MPI* myocardial performance index.

*: p < 0.05.

Upon returning to Earth, sitting HR increased at both 3 and 8 days post-landing (p < 0.001, d = 1.40 and p < 0.001, d = 1.09, respectively), compared to the last measurement onboard the ISS. HR was also elevated compared to the baseline in sitting position for both measurements in the recovery period (p = 0.003, d = 0.97 and p = 0.037, d = 0.62, respectively). After returning to Earth, PEP, PEPi, PEP/LVET, and MPI reverted to their baseline values.

The results were different when comparing in-flight measurements to the baseline measured in supine position, with an increase in HR after five months (p = 0.013, d = 0.86). Conversely to the comparison with the sitting baseline, the PEP/LVET ratio was elevated during the first week in-flight compared to the supine baseline (p = 0.049, d = 0.45).

At both 3 and 8 days post-flight, supine HR was higher compared to baseline values in the same position (p < 0.001, d = 1.28; p = 0.002, d = 0.70, respectively). No significant changes were observed for any of the other parameters when using the supine position as a baseline.

**Fig. 1:**
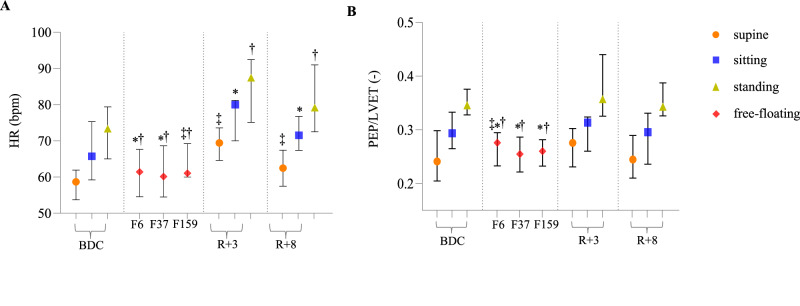
Longitudinal evolution of some cardiac time intervals features throughout the study. **A** heart rate (bpm); **B** ratio of pre-ejection period and left ventricular ejection time (-). Measurements are taken at baseline (BDC), during the flight on day 6 ± 2, 37 ± 4, and 159 ± 4 (F6, F37, and F159, respectively), and during recovery at 3 and 8 days post-flight (R + 3 and R + 8, respectively). Results are presented as median [first quartile; third quartile]. ‡: p < 0.05 compared to supine baseline position, *: p < 0.05 compared to sitting baseline position, †: p < 0.05 compared to standing baseline position. Statistical analysis was performed using a mixed-effects model with Holm-Sidak correction for multiple comparisons.

The results of the comparisons with different baseline positions are illustrated in Fig. 1A, B for HR and PEP/LVET, respectively. In addition, the changes occurring during the transition between the supine and the standing position are available in Supplementary Information (Supplementary Table 1), reporting no differences post-flight versus pre-flight.

### Cardiac mechanical performance and hemodynamics

**Fig. 2:**
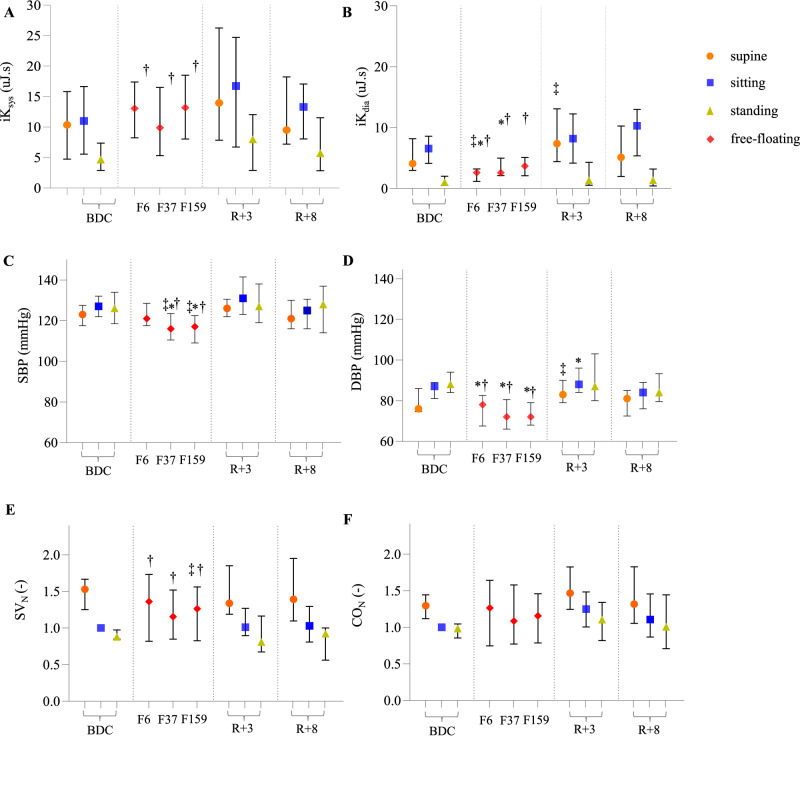
Longitudinal evolution of the mechanical performance and hemodynamic parameters throughout the study. **A** integral of kinetic energy measured during systole; **B** integral of kinetic energy measured during diastole; **C** systolic blood pressure; **D** diastolic blood pressure; **E** stroke volume computed with Kubicek formula normalized by the baseline sitting value; **F** cardiac output computed with Kubicek formula normalized by the baseline sitting value. Measurements are taken at baseline (BDC), during the flight on day 6 ± 2, 37 ± 4, and 159 ± 4 (F6, F37, and F159, respectively), and during recovery at 3 and 8 days post-flight (R + 3 and R + 8, respectively). Results are presented as median [first quartile; third quartile]. Statistical analysis was performed using a mixed-effects model with Holm-Sidak correction for multiple comparisons. ‡: p < 0.05 compared to supine baseline position, *: p < 0.05 compared to sitting baseline position, †: p < 0.05 compared to standing baseline position.

Cardiac mechanical performance and hemodynamics were also assessed, beginning with comparisons to the sitting baseline position. Table 2 presents an overview of the changes in systolic and diastolic integrals of SCG kinetic energy (iK), blood pressure, stroke volume normalized to the baseline sitting value (SV_N_), and cardiac output normalized to the baseline sitting value (CO_N_) at the different time points. Systolic brachial blood pressure (SBP) was lower compared to the sitting baseline position during both the first and fifth month in-flight (p < 0.001, d = 1.43 and p < 0.001, d = 1.47, respectively), and diastolic brachial blood pressure (DBP) was lower in all the three in-flight measurements (p = 0.012, d = 1.25; p < 0.001, d = 1.45; p = 0.002, d = 1.50 after one week, one month, and five months, respectively), compared to baseline values in sitting position. Post-flight, sitting DBP was higher than the sitting baseline three days after returning to Earth (p = 0.049, d = 0.66). Figure 2C, D illustrate the changes in SBP and DBP, respectively.

**Table 2:** Evolution of cardiac mechanical performance and hemodynamic parameters throughout the study

	BDC sitting n = 17	F6 n = 17	F37 n = 17	F159 n = 17	R + 3 sitting n = 15	R + 8 sitting n = 17
SBP (mmHg)	127 [122, 132]	121 [117, 128]	116 [110, 123]*	117 [109, 122]*	131 [123, 141]	125 [116, 130]
DBP (mmHg)	87 [81,89]	78 [67,82]*	72 [66,80]*	72 [68,79]*	88 [84,96]*	84 [79,93]
SV_N_ (-)	*1.00*	1.36 [0.82, 1.73]	1.15 [0.85, 1.52]	1.26 [0.82, 1.56]	1.01 [0.90, 1.27]	1.03 [0.81, 1.29]
CO_N_ (-)	*1.00*	1.27 [0.75, 1.58]	1.09 [0.77, 1.58]	1.16 [0.79, 1.46]	1.25 [1.00, 1.48]	1.11 [0.87, 1.46]
iK_sys_ (uJ.s)	11.01 [5.57, 16.67]	13.07 [8.24, 17.39]	9.89 [5.32, 16.52]	13.19 [8.04, 18.50]	16.96 [13.08, 24.99]	13.30 [8.05, 17.07]
iK_dia_ (uJ.s)	6.91 [4.18, 10.51]	2.64 [1.20, 3.23]*	2.61 [2.15, 5.12]*	3.72 [2.11, 5.12]*	9.76 [4.85, 32.30]	10.3 [5.39, 13.00]
iK_sys__i (%)	56.7 [50.1, 69.6]	85.4 [80.2, 89.5]*	75.4 [65.8, 82.1]*	77.6 [72.0, 84.8]*	62.8 [53.6, 70.9]	58.5 [50.5, 65.2]
iK diastolic ratio (-)	0.85 [0.79, 0.95]	0.68 [0.81, 0.88]	0.89 [0.77, 0.95]	0.86 [0.74, 0.91]	0.83 [0.49, 0.92]	0.75 [0.56, 0.89]

Measurements are taken at baseline (BDC), during the flight on day 6 ± 2, 37 ± 4, and 159 ± 4 (F6, F37, and F159, respectively), and during recovery at 3 and 8 days post-flight (R + 3 and R + 8, respectively). Comparisons are made relative to baseline values in the sitting position using a linear mixed-effects model with Holm-Sidak correction for multiple comparisons. Results are presented as median [first quartile; third quartile]. Italics: 1.00 by definition (normalized values).

*SBP* systolic blood pressure, *DBP* diastolic blood pressure, *SV*_N_ stroke volume computed with Kubicek formula normalized by the baseline sitting value, *CO*_N_ cardiac output computed with Kubicek formula normalized by baseline sitting value, *iK*_sys_ integral of seismocardiography (SCG) kinetic energy during systole, *iK*_*dia*_ integral of SCG kinetic energy during diastole, *iK*_sys__*i* ratio of iK_sys_ and the integral of SCG kinetic energy during the whole cardiac cycle, *iK diastolic ratio*: difference between the integral of SCG kinetic energy during late and early diastole, normalized by iK_dia._

*: *p* < 0.05.

SV_N_ and CO_N_ were unchanged at all time points when they were compared to the baseline sitting values. The evolution of these two features is illustrated in Fig. 2E, F, respectively.

Although the absolute values of systolic iK remained relatively stable throughout the study when compared to the sitting baseline position (Fig. 2A), this was not the case for the systolic kinetic energy normalized to the overall kinetic energy throughout the cardiac cycle (iK_sys__i), which increased in-flight (p < 0.001, d = 2.15; p = 0.003, d = 1.56; and p = 0.001, d = 1.46 for the first week, first month, and fifth month, respectively). This was mainly caused by the evolution of the diastolic kinetic energy (iK_dia_) in-flight (see also Fig. 2B). Indeed, iK_dia_ greatly decreased in-flight, when compared to the sitting position (p < 0.001, d = 1.44 and p = 0.014, d = 1.16 for the first week and the first month, respectively). Meanwhile, the diastolic ratio, representing the distribution of energy between passive and active filling, did not change. All the features based on SCG kinetic energy were back to their baseline values as early as three days post-flight.

Compared to the supine baseline position, SV_N_ was lower in the fifth month in-flight (p = 0.027, d = 0.73), while CO_N_ remained unchanged. SBP decreased during both the first and the fifth month in-flight (p = 0.027, d = 0.88 and p = 0.019, d = 0.92, respectively). iK_dia_ was reduced only after one week in-flight (p = 0.008, d = 1.00), while all the other features based on SCG kinetic energy were preserved.

At three days post-flight, the supine DBP was higher than the supine baseline position (p = 0.016, d = 0.68), which was also the case for iK_dia_ (p = 0.028, d = 0.76).

## Discussion

This study suggests that the cosmonauts involved in long-duration missions onboard the ISS and assigned to the current routine countermeasures are subject to relatively small changes in cardiac time intervals and mechanical performance. Most in-flight features computed from ICG and SCG demonstrate no or subclinical changes depending on the reference position used for baseline measurements. When they occur, these subclinical changes are often lower than those occurring during the transition from a supine to a standing position on the Earth. In addition, the recovery after flight is relatively quick, with most features already back to their baseline values three days post-flight, which is also true for the effect of the orthostatic stress on the resting supine values. Besides this, these observations demonstrate the interest in using wearable technologies such as SCG and ICG in extreme environments, where they can be used to provide rapid, non-invasive assessments, allowing for more frequent measurements.

When comparing in-flight data to the supine baseline, a decrease in SV accompanied by an increased HR was observed as a compensatory mechanism to maintain CO after five months of microgravity. Our findings align with the understanding that, over prolonged periods in microgravity, the cardiac function may slightly be altered, resulting in a lower SV compensated by a positive chronotropic effect, likely driven by enhanced sympathetic nervous system activity. As a comparison, some studies have indicated a decrease in resting SV among cosmonauts after a 5-month mission^5^, while other studies reported no long-term changes^6,8^, and some others found an increase^7^. Similar discrepancies are also seen in the assessment of resting HR, with some studies documenting an increase in HR in microgravity^9^, others suggesting no change^7,10^, and others a decrease in HR^11^. These differences in findings can primarily be attributed to the risk of type II errors in studies involving only a small number of participants, as well as to the selection of the baseline reference position^33^. Indeed, when comparing in-flight to baseline sitting measurements, in-flight SV was found to be stable, and HR to be lower than baseline in the early phases of the flight. In some cases, opposite conclusions may be drawn depending on the reference position used.

When comparing in-flight results to the sitting baseline, it appears that myocardial performance may have been enhanced, as indicated by decreases in PEP, PEP/LVET, and MPI. However, compared to the supine position, MPI did not change, while both PEP and PEP/LVET increased during the first week before returning to preflight values. These observations may reflect a higher cardiac preload in supine position and in weightlessness than in sitting position. Few studies have examined cardiac time intervals during spaceflight, and these studies typically involved smaller sample sizes. Slightly contrasting with our findings, one study reported that LVETi based on finger pulse waveform was higher in-flight than the sitting baseline, while it was relatively similar to the supine baseline^34^.

Besides this, a decrease in resting SBP was observed in-flight compared to both supine and sitting baseline positions, even though DBP decreased only compared to the measurements performed in sitting position. Interestingly, this decrease in SBP was not observed in previous studies recruiting a lower number of participants^6,11,35^.

Onboard the ISS, a range of countermeasures is currently used to mitigate cardiovascular deconditioning, including resistive exercise^36^, aerobic exercise^37^, and lower body negative pressure devices^38^. The evolution of the features measured in this study seems to indicate that these countermeasures effectively preserved a cardiovascular state that was relatively similar to the one on Earth throughout the mission of the cosmonauts onboard the ISS. Overall, the different changes observed were subclinical and lower than those occurring when changing position on Earth.

The results of this study can also be compared to those of head-down bed rest (HDBR), a widely used analog of microgravity that allows the evaluation of the impact of countermeasures. Compared to supine values and in the absence of effective countermeasures, an increase in HR and a decrease in SV have already been evidenced during HDBR^39^. Conversely, with adapted countermeasures, these changes could be attenuated or even reversed^40–42^. Regarding LVETi, conflicting results have been observed^43,44^, with even one study reporting different results for LVET between two successive campaigns^19^. Besides this, it has been shown that PEP/LVET was increased compared to the supine baseline after 21 days of HDBR^43^, which is similar to what was transiently observed here before a return to baseline supine values after one and five months in space.

SCG kinetic energy has been measured in only one HDBR study. In both an exercise and a control group, it was observed that iK_sys_ decreased after 5 days of HDBR before going back to baseline supine values, while iK_dia_ decreased throughout the exposure to HDBR^40^. Even if there are some signs that SCG kinetic energy is correlated with SV^26^, here, the evolution of iK_sys_ and iK_dia_ did not follow the one of SV measured by ICG. This may be due to differences in the position of the SCG sensor (apex here versus sternum in other studies), changes in the orientation of the heart in microgravity, as well other hemodynamic changes that are specific to this environment. However, it should also be noted that the Kubicek formula, providing the value of SV based on the ICG amplitude and LVET, has not been validated in space or HDBR. This represents a limitation of the current study, as fluid distribution, electrode contact, and thoracic impedance properties may be altered in space, potentially affecting the accuracy of SV estimation. As such, results based on SV should be interpreted with appropriate caution.

Previously, it has been stated that exposure to HDBR induced a hemodynamic state that lay between the upright and supine positions^4^. Our findings indicate that this is also relatively true in actual microgravity. However, to better assess the fine changes occurring in microgravity, we recommend reporting both the baseline values in the supine and sitting positions. 24 h records could also be used to better compare the typical cardiovascular status during a day on the Earth versus in space. This is crucial for efficient risk management and developing effective countermeasures to protect crew health during prolonged space missions.

Numerous reports show that, even though a new hemodynamic state is established in weightlessness, this new state is not compatible with a quick return to terrestrial conditions and can lead to orthostatic intolerance^45^. In the present study, at three days post-flight, HR and DBP were higher than their baseline values in all tested positions, which agrees with other observations about an increase or a trend towards an increase of HR during the recovery phase, even with in-flight countermeasures^6,11,46,47^. This confirms the numerous reports indicating an increased resting sympathetic activity in the phase of early post-flight recovery^48^. Besides this, all the other post-flight resting values in supine, sitting, and standing positions showed a return to their baseline values as early as three days post-flight. At 8 days post-flight, the recovery seemed also completed for HR and DBP.

Regarding SV, in the present study, the results obtained post-flight with wearable cardiac monitoring are very similar to those obtained by MRI in the same cosmonauts^49^, but also among American astronauts^50^, a few days post-flight. Similar observations were also reported after HDBR studies, where SV was back to baseline following 4 days of recovery, even in the absence of effective countermeasures, while HR was generally elevated^39^. Here, interestingly, even though HR was increased and SV was unchanged three days post-flight, only a trend for an increase in CO could be observed (p = 0.067 and p = 0.065 in the supine and sitting positions, respectively).

The absence of changes in the PEP/LVET ratio and PEPi in the present study is consistent with the results of different HDBR studies. One HDBR study comparing baseline values to those 2 days after exposure to simulated microgravity showed no change in the PEP/LVET ratio^43^. Meanwhile, in another HDBR study, an increase of PEPi was visible in a control group early after HDBR, while no changes were observed in a group performing lower-body negative pressure the last week before the end of exposure to HDBR^51^. When comparing these findings with the absence of post-flight changes in PEPi among Russian cosmonauts, it is important to mention that the latter are also assigned to a lower-body negative schedule prior to their return to Earth, in addition to their exercise countermeasure protocol.

Overall, these results suggest that the combination of the routine exercise countermeasure, as well as the additional activities specifically planned prior to landing and immediately after landing, allow for a quick readaptation of the cardiovascular status to the terrestrial gravity.

This study did not reveal large differences between pre- and post-flight records for sitting and supine positions, which are traditionally used as reference positions. While standing recordings also showed similar pre- and post-flight values, this position may have been more sensitive to microgravity-induced deconditioning. Indeed, a large proportion of individuals assigned to long-duration spaceflight experiences orthostatic intolerance on return to terrestrial gravity^45^. However, in this study, as shown on Figs. 1 and 2, as well as supplementary Table 1, the effect of changing posture from supine to standing remained largely consistent across all time points studied. While this observation may be surprising, it should also be noted that 2 cosmonauts could not be evaluated in standing position at the first post-flight data point. This may have introduced a bias in the statistical analyses. Besides this, as already mentioned, lower-body negative pressure, together with saline loading and compression garments were certainly used by the Russian crew members prior to, during, or immediately after landing^34^. These types of countermeasures have already shown their benefits in helping with orthostatic tolerance^52,53^.

It has been demonstrated that individuals who show orthostatic intolerance have a blunted increase in norepinephrine levels during standing^54^, which may contribute to orthostatic hypotension^55^. To finely assess these abnormal responses to orthostatic stress, additional features based on HR variability may be more sensitive than the ones presented here. In addition, the assessment of SV variability may also provide insightful information regarding a potentially hypovolemic status of the crew members after spaceflight. Even if they are not the subject of this manuscript, these features could be computed with devices similar to the one used in this research.

It is important to consider certain limitations when interpreting these findings. One primary limitation is the impact of changes in the position of the heart on the reliability of SCG measurements. As the heart’s position and orientation can shift due to microgravity effects, the placement of the sensor may need optimization to align accurately with these changes. This limitation could be partially overcome by measuring SCG in three dimensions rather than on only one axis^40,56^. While this manuscript focuses on ECG, SCG, and ICG signals, incorporating BCG data might also provide additional insights by capturing the mechanical activity associated with blood ejection.

Furthermore, the analysis of spaceflight data presents inherent challenges due to the typically small sample size. While 17 participants is considered a relatively large sample in this field, small cohorts limit the statistical power of the findings, suggesting that larger datasets are essential for a more comprehensive understanding of cardiac function in these extreme conditions.

Another limitation is the uncertainty regarding the individual protocol adopted to help manage fluid redistribution after landing in each cosmonaut (e.g., saline infusions or compression socks). The use of such interventions may have influenced the post-flight results, including the orthostatic stress caused by the postural changes from supine to standing. Another factor to consider is the possibility that crew members did not strictly adhere to the countermeasure program, which could have contributed to inter-individual variability^34^.

Additionally, three subjects underwent their first in-flight measurement onboard the ISS 2–3 days after launch. This early timing may have impacted the results of these subjects for the first week in-flight data point. Indeed, the acute physiological changes due to microgravity usually occur in the first 48 h^57^.

Despite these limitations, the use of advanced wearable technologies like SCG and ICG remains a promising approach for acquiring valuable data on cardiac function, which is crucial for evaluating the overall cardiovascular health and efficacy of countermeasures in the context of human spaceflights. This could pave the way for more personalized and continuous monitoring, enabling closer follow-up in future missions.

In summary, despite variations depending on the baseline position used as a reference, most cardiovascular features remained stable and within subclinical ranges throughout the spaceflight. In addition, most of these features returned to their baseline values three days after landing, even in the standing position. The comparison with terrestrial analogs of microgravity, showing larger changes in the absence of countermeasures, suggests that the countermeasure regime applied to the cosmonauts is effective. Data from wearable technologies, such as SCG and ICG, have the potential to enable frequent, non-invasive measurements that ensure consistent data collection in extreme environments and different positions. While there may be a debate regarding the optimal position to be used as a reference on the ground, to enhance the robustness of terrestrial comparisons we recommend that, whenever possible, future research compares weightlessness observations to both supine and sitting positions on the Earth.

## Methods

### Participants

In this paper, we present the results from 17 cosmonauts (16 men, 1 woman) with (median [first quartile; third quartile]): age of 44 [41; 50] years, body weight of 83 [76; 89] kg, and body height of 178 [173; 180] cm at the time of their pre-flight record. They stayed 185 [172; 270] days onboard the ISS, with 13 (1 woman) of them assigned to a 6-month mission and 4 of them assigned to a 1-year mission. During their time in space, the cosmonauts followed their normal schedule, including countermeasures based on physical exercise. Informed consent was obtained from each participant prior to starting the study. Additionally, they were made aware of their right to withdraw from the study at any time.

As part of their countermeasure regimen, participants followed a structured exercise protocol consisting of twice-daily physical training, for a total of 2.5 h per day, using all available onboard exercise equipment. The training program included static exercises (loading suits), dynamic activities (walking, running, jumping, strength training, cycling), and inertial-impact actions. It followed 4-day training microcycles, consisting of three loading days and one day of active rest^34^. On the treadmill, they used a high-intensity interval approach on the first day, a medium intensity the second day, and a lower intensity on the third day. Approximately 30 to 40 days before the end of their mission, they transitioned to the prescribed training protocols for the final phase. With few exceptions, this phase required locomotor training sessions to be performed twice daily, following the regimen designated for the first day of the microcycle^34^.

### Material

**Fig. 3:**
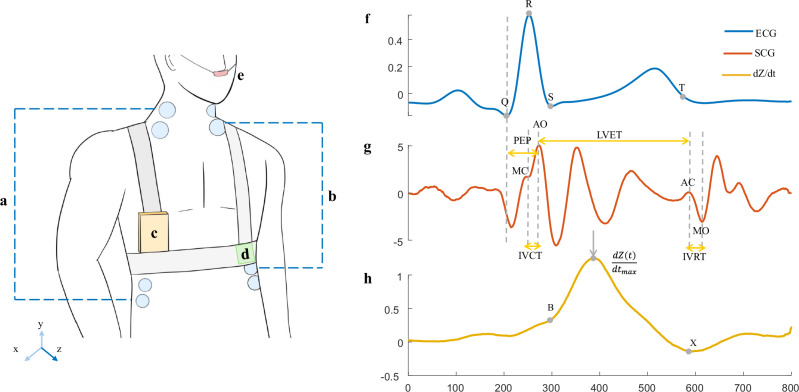
Schematic representation of the different elements of CARDIOVECTOR. **a** Impedance cardiography (ICG) current electrodes; **b** ICG measuring electrodes; **c** Main unit (connection and amplification); **d** Seismocardiography (SCG) sensor at the cardiac apex (dorsoventral linear accelerations); **e** Plethysmography sensor (nasal thermistor); **f** ECG signal and its fiducial points; **g** SCG signal, its fiducial points (MC Mitral Closure, AO Aortic Opening, AC Aortic Closure, MO Mitral Opening), and cardiac time intervals (PEP Pre-ejection Period, LVET Left Ventricular Ejection Time, IVCT Isovolumetric Contraction Time, IVRT Isovolumetric Relaxation Time); **h** ICG signal and its fiducial points (B point: onset of left ventricular ejection, C point or $\frac{{\rm{dZ}}({\rm{t}})}{{{\rm{dt}}}_{\max }}$ : the maximum rate of impedance change during systole, and X point: the onset of aortic valve closure).

During the protocol, ECG, SCG, ICG, and respiration were measured using the CARDIOVECTOR device (IBMP, Zelenograd, Russian Federation), illustrated in Fig. 3. These data were collected as part of the larger CARDIOVECTOR and CARDIOVECTOR-3 experiments conducted onboard the ISS. While the primary goal of these experiments was to investigate multi-dimensional ballistocardiography in microgravity, this manuscript focuses specifically on data from the ECG, ICG, and SCG channels. Eight participants were evaluated using the first version of the device, while nine were assessed with the second version. The primary distinction between the two versions is that the second version allows for the recording of an additional signal from the lower back, which, however, is not the focus of this study. The standard nomenclature was used^14^: for the SCG signal, the only acquired axis is z, representing the anteroposterior (dorsoventral) direction, and recordings were taken at the apex. The ICG setup incorporated 8 electrodes, including 4 outer (current electrodes) and 4 inner electrodes (measuring electrodes). The outer electrodes are used to transmit the current through the thorax, while the inner electrodes, positioned between the current electrodes, measure the voltage changes resulting from the current flow. All signals were acquired at a sampling rate of 2000 Hz. Additionally, SBP and DBP were measured prior to each recording. On the ground, all supine measurements were performed on the same bed to ensure consistency.

### Data collection protocol

**Fig. 4:**
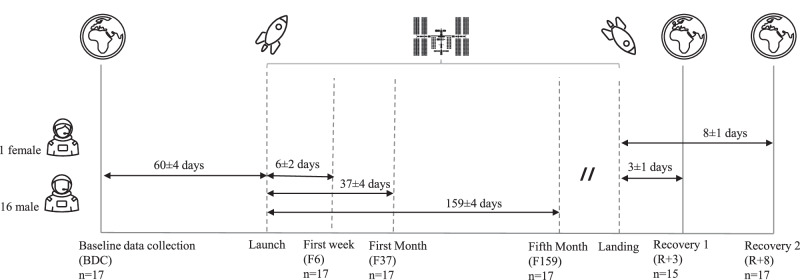
Seismocardiography and impedance cardiography data collection protocol. Measurements included in this study were conducted pre-flight (BDC; 60 ± 4 days before launch), post-flight (R + 3, R + 8; 3 ± 1 and 8 ± 1 days after return, respectively), and in-flight during microgravity at three time points: the first week (F6; 6 ± 2 days), the first month (F37; 37 ± 4 days), and the fifth month (F159; 159 ± 4 days). The pre-flight and post-flight sessions included measurements in supine, sitting, and standing positions.

This study was carried out on cosmonauts assigned to missions onboard the ISS and consisted of a longitudinal evaluation of features extracted from SCG and ICG signals. One recording was made pre-flight, two post-flight, and one per month in-flight. The data points considered in this paper consist of the pre- and post-flight records (60 ± 4 days before flight, 3 ± 1 and 8 ± 1 days after flight, respectively), as well as the ones collected during the first week, first month, and the fifth month in microgravity (6 ± 2, 37 ± 4, and 159 ± 4 days, respectively) (See Fig. 4). The first in-flight data point was planned late enough for the acute microgravity-related adaptations to be over, but before the long-term adaptations could arise, in the context of daily routine exercise countermeasures.

At each acquisition session, subjects were asked to follow a guided respiratory protocol, from which this work will analyze only a subpart. The complete protocol included a part consistent across both terrestrial (sitting position) and space (free-floating position) measurements: 1) 5 minutes of uncontrolled breathing; 2) 3 minutes of 5-second breathing cycles; 3) 3 minutes of 10-second breathing cycles; 4) maximal (up to 3 minutes) end-inspiratory apnea; 5) maximal (up to 2 minutes) end-expiratory apnea, with 1 minute of rest between each phase. In addition, in the pre- and post-flight measurements, participants performed two additional tasks: 6) 10 minutes of uncontrolled breathing in supine position; 7) 10 minutes of uncontrolled breathing in standing position. In this study, we focused on uncontrolled normal breathing, including the 5-minute acquisition performed in free-floating in space or in a sitting position on the ground (step 1), as well as the 10-minute records performed both in supine and standing positions on the ground (steps 6 and 7). For more details, see Supplementary Fig. 1.

The study protocol complied with the Declaration of Helsinki and was approved by the Biomedical Ethics Committee of the Institute of Biomedical Problems of the Russian Academy of Sciences from the 25th of May 2012 (CARDIOVECTOR 1) and from the 20th of June 2018 (CARDIOVECTOR 3), the Erasme University Hospital Ethics Committee (P2017/332/CCBB406201732664), as well as the medical boards of all partners of the ISS program and Human Research Multilateral Review Board.

### Data analysis

Definitions and physiological relevance of the parameters described below are summarized in Supplementary Table 2.

The kinetic energy analysis based on SCG signal was performed in MATLAB (R2023a, MathWorks). The computation of kinetic energy, based on SCG, started with downsampling of both the ECG and SCG signals at 1000 Hz, followed by bandpass filtering. The ECG signal was processed using a 4th-order Butterworth bandpass filter with a 0.5–100 Hz passband. The SCG signal was processed using the same type and order of filter but with a 5–25 Hz passband.

Subsequently, the R waves, marking each cardiac beat, were automatically detected within the ECG signal. These detections underwent a visual check for accuracy, with corrections manually applied if necessary. The identified R waves served as fiducial markers to separate the individual cardiac beats. Then, the ensemble average was computed by aligning and averaging the ECG and SCG signals from multiple cardiac cycles. Complementary information regarding the computation of the ensemble average is reported elsewhere^58^.

Then, the kinetic energy transmitted to the SCG sensor along the z-axis by the cardiac activity was computed based on the sensor’s inertial parameters. Finally, the iK was computed across the whole cardiac cycle, as well as across the systolic (from Q to end of T wave) and diastolic (from the end of the T wave to P wave and from P wave until the next Q wave, for early and late diastole, respectively) phases:

1 $${{iK}}_{{desired\; phase}}={\int }_{{desired\; phase}}\frac{1}{2}m.{{V}_{z}(t)}^{2}.{dt}$$

where m is the mass of the sensor and V_z_ is the velocity in the z (anteroposterior) direction, which is computed by integrating the acceleration signal. The computation of the kinetic energy features is reported in more detail elsewhere^26^.

The percentage of systolic iK (iK_sys__i (%)) is defined as the ratio of iK_sys_ to the total iK (over the entire cardiac cycle), expressed as a percentage. The diastolic ratio (iK diastolic ratio (-)) is defined by Eq. (2) as the difference of the iK between the TP and PQ phases (representing passive and active filling, respectively), normalized by iK during the complete diastolic phase (TQ = TP + PQ)^59^.

2 $${iK\; diastolic\; ratio}=\frac{{{iK}}_{{TP}}-{{iK}}_{{PQ}}}{{{iK}}_{{TP}}+{{iK}}_{{PQ}}}$$

The integral of kinetic energy derived from SCG serves as a non-invasive proxy for cardiac mechanical function and has been associated with stroke volume and ejection fraction in both healthy individuals and patients^59^.

SCG-based cardiac time intervals were computed by first identifying the onset of ventricular depolarization, marked by the Q wave on the ECG signal. In contrast, the opening of the aortic valve is a mechanical event that cannot be directly detected on the ECG. While it can be derived by changes in thoracic electrical impedance (ICG)^60^, measures based on chest vibrations (SCG) have been shown to be more reliable^23^.

The assignment of peaks and valleys on the SCG to the specific cardiac events was done after obtaining ensemble-median signals on each channel, based on the illustrations provided by Crow et al.^61^. To locate the fiducial points, we focused on specific windows set after the R peak and around the T wave of the ECG. These search intervals were chosen as defined in relevant literature^22^. The systolic window was defined as the interval from 25 to 75 ms after the R peak, while the diastolic window was defined as a 60 ms segment centered on the end of the T wave. In the systolic window, we identified the highest peak in the SCG signal as the aortic valve opening (AO) point, while the last peak before this AO was designated as the mitral valve closure (MC) point. For the diastolic events, the first peak in the SCG signal within the diastolic window was identified as aortic valve closure (AC), and mitral valve opening (MO) was assumed to occur at the subsequent valley. These automatic detections were then subject to visual inspection and manually corrected when necessary.

After finding these fiducial points, the PEP and LVET were computed as the interval between Q and AO, and the interval between AO and AC, respectively. PEP can serve as an indicator of myocardial contractility when left ventricular end-diastolic pressure and afterload remain constant^62^. On the other hand, LVET reflects the duration of blood ejection from the left ventricle into the aorta and is affected by various factors, including cardiac dysfunction, with a shortening in pathologic conditions such as heart failure^63^. Following this, the ratio PEP/LVET was computed. Changes in this index may be less impacted by preload and afterload, and an increase in PEP/LVET can be indicative of diminished cardiac contractility^64^. As suggested by^65^, the normalized features PEPi and LVETi were computed as follows:

3 $${LVETi}=\left\{\begin{array}{ll}1.7\times {HR}+LVET\,\left({males}\right)\\ 1.6\times {HR}+LVET\,\left({females}\right)\end{array}\right.$$

4 $${PEPi}=0.4\times {HR}+{PEP}$$

where HR is measured in bpm, and LVET and PEP are measured in milliseconds.

Two additional cardiac time intervals were computed as described by di Rienzo et al.^64^: the isovolumic contraction time (IVCT, from MC to AO) and the isovolumic relaxation time (IVRT, from AC to MO).

Furthermore, MPI, also known as the Tei-index, was derived from these parameters^64^, and defined as:

5 $${MPI}=\frac{{IVCT}+{IVRT}}{{LVET}}$$

SV was estimated using a formula introduced by Kubicek^15^ based on the ICG signal.

6 $${{SV}}_{{Kubicek}}=\frac{\rho {L}^{2}}{{Z}_{0}^{2}}\frac{{dZ}(t)}{{{dt}}_{\max }}{LVET}$$

where, ρ is the electrical resistivity of blood (Ω.cm), L is the mean distance between the two measuring electrodes (cm), Z(t) is the instantaneous impedance (Ω), Z_0_ is the basal impedance between the inner (measuring) electrodes (Ω), $\frac{{dZ}(t)}{{{dt}}_{\max }}$ is the maximum rate of impedance change during systole (C point amplitude in Ω.s^-1^, as shown in Fig. 3)^15^.

On the ICG signal, the LVET is usually defined as the time between the B point (corresponding to aortic valve opening) and the X point (corresponding to aortic valve closure). However, their automated detection remains challenging due to breathing, high-frequency noise, body movements or muscle contractions, etc. These factors alter the shape of the dZ/dt signal, resulting in inter- and intra-subject variability^60^. Therefore, to achieve better precision, in this article, the decision was made to use formula (6) based on LVET measured from the SCG signal.

As the parameter L was inaccessible to the authors, the analysis focuses on relative changes rather than absolute values. Stroke volume was thus normalized to the baseline sitting position value, referred to as SV_N_.

Then CO was computed as follows:

7 $${CO}={{SV}}_{{Kubicek}}\times {HR}$$

CO was similarly normalized to its baseline value in sitting position, to define CO_N_.

### Statistics

The statistical analysis was conducted using GraphPad Prism version 9.5.1 (GraphPad Software, San Diego, CA, United States). Two records obtained at the first post-flight time point were discarded due to insufficient quality. Consequently, both for the results reported in sections 2.1 and 2.2, mixed-effects models were fitted with time as a fixed effect and participant as a random effect. Adjustments for multiple comparisons were applied using the Holm-Sidak correction method. Effect sizes were computed and reported using Cohen’s d, where d = 0.2 reflects a small effect, d = 0.5 a medium effect, and d = 0.8 a large effect^66^. Statistical significance was established at a p-value below 0.05. Results are presented as the median [first quartile; third quartile].

## Supplementary information

Supplementary information

## Data Availability

Data are not available. Requests to access datasets should be directed to the Institute of Biomedical Problems (Russian Academy of Sciences).
