# Supplementary material for: Evidence of effective cardiovascular countermeasures during spaceflights: insights from wearable monitoring
Source: NPJ Microgravity. 2025 Sep 30;11:68. doi: 10.1038/s41526-025-00522-8 (PMC12484674; doi:10.1038/s41526-025-00522-8)
Supplement: Supplementary file 1 — Supplementary information [file 41526_2025_522_MOESM1_ESM.pdf]

Supplementary Information

Supplementary note 1: Respiratory protocol

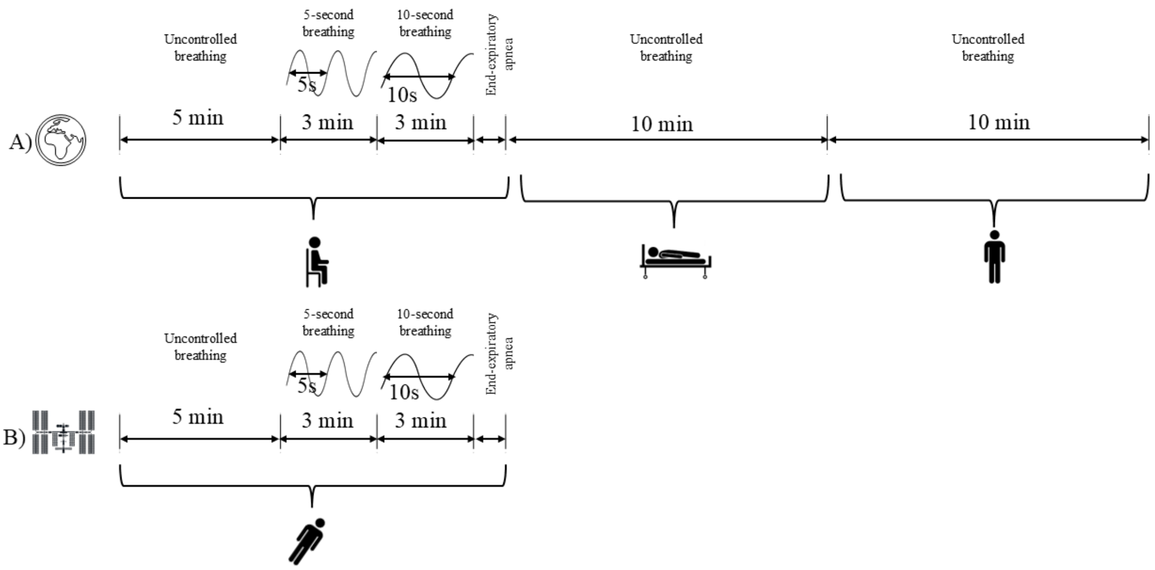

Supplementary Figure 1. Respiratory protocol performed by the crew A) in various body positions on Earth B) in free-floating condition onboard ISS.

Supplementary note 2: Orthostatic stress

Preflight, the transition from supine to standing induced an increase in HR ($p<0.001$, $d=1.83$) and a decrease in SV_N ($p<0.001$, $d=2.41$). After spaceflight the effect of this orthostatic stress on these features remained unchanged. The exact same trend was also observed for all the other tested features (See Supplementary Table 1).

Supplementary Table 1. Effect of changing from supine to standing position, before and after flight. HR: heart rate; PEP: pre-ejection period; LVET: left ventricular ejection time; SBP: systolic blood pressure; DBP: diastolic blood pressure; SV_N: stroke volume computed with Kubicek formula normalized by the baseline sitting value; CO_N: cardiac output computed with Kubicek formula normalized by the baseline sitting value;; iK_{sys}: integral of seismocardiography (SCG) kinetic energy during systole; iK_{dia}: integral of SCG kinetic energy during diastole; iK_{sys}_i ratio of iK_{sys} and the integral of SCG kinetic energy during the whole cardiac cycle. Measurements are taken at baseline (BDC), and during recovery at 3 and 8 days post-flight (R+3 and R+8, respectively). Results are presented as median [first quartile; third quartile]. Statistical analysis was performed using a mixed-effects model with Holm-Sidak correction for multiple comparisons. No statistical significance ($p<0.05$) was observed when R+3 and R+8 were compared to baseline.

	BDC<br>n=17	R+3<br>n=15	R+8<br>n=17
HR (bpm)	+17 [10, 19]	+16 [12, 22]	+20 [11, 24]
PEP (ms)	+11 [7, 20]	+12 [6, 21]	+11 [-1, 20]
LVET (ms)	-56 [-78, -38]	-57 [-78, -38]	-62 [-81, -45]
PEP/LVET (-)	+0.09 [0.05, 0.16]	+0.12 [0.08, 0.15]	+0.12 [0.04, 0.15]
SBP (mmHg)	+5 [-2, 9]	+4 [-4, 8]	+7 [-3, 11]
DBP (mmHg)	+9 [3, 13]	+6 [-3, 12]	+7 [0, 13]
SV_N (-)	-0.60 [-0.83, -0.48]	-0.70 [-0.93, -0.60]	-0.52 [-0.94, -0.29]
CO_N (-)	-0.35 [-0.61, -0.27]	-0.49 [-0.73, -0.37]	-0.18 [-0.57, -0.13]
iK_{sys}_i (%)	+13.64 [-7.43, 22.39]	+14.94 [0.69, 24.34]	+21.05 [6.28, 24.60]
iK_{sys} (uJ.s)	-4.04 [-10.29, -0.95]	-5.94 [-14.91, +0.77]	-4.6 [-10.75, -0.18]
iK_{dia} (uJ.s)	-3.42 [-7.32, -1.35]	-4.80 [-8.42, -2.28]	-3.57 [-10.11, -1.28]

Supplementary note 3: Summary of cardiovascular parameters analyzed in this study

Supplementary Table 2. Summary of cardiovascular parameters analyzed in this study, including definitions and their physiological meaning.

Parameter	Definition	Physiological Meaning
PEP (ms)	Pre-ejection period	Time from electrical depolarization to valve opening; negatively correlated with contractility
LVET (ms)	Left ventricular ejection time	Duration of blood ejection; influenced by preload and afterload
PEP/LVET (-)	Ratio of PEP to LVET	Contractility index; lower values suggest stronger contractility
MPI (-)	Myocardial Performance Index (Tei index)	Assesses left ventricular systolic and diastolic function. An elevated Tei index reflects impaired overall cardiac function.
IVCT (ms)	Isovolumetric contraction time	Index of myocardial contractility
IVRT (ms)	Isovolumetric relaxation time	Index of heart relaxation; closely linked to contractility
iK_{sys} (uJ.s)	Integral of kinetic energy during systole	Mechanical action during ventricular contraction
iK_{dia} (uJ.s)	Integral of kinetic energy during diastole	Mechanical action during ventricular filling
iK_{sys}_i (%)	Percentage of iK_{sys} over the entire cardiac cycle	Evaluates systolic contribution to overall heart activity
iK diastolic ratio (-)	Ratio of early to late diastolic kinetic energy	Reflects balance between passive (early) and active (late) filling
